# The effect of rotational velocity on rotational traction across a range of artificial turf surface systems

**DOI:** 10.1038/s41598-023-48134-0

**Published:** 2023-12-07

**Authors:** Harry McGowan, Paul Fleming, Jae-Hwi Pak, David James, Steph Forrester

**Affiliations:** 1 School of Architecture, Building and Civil Engineering, Loughborough University, Loughborough, UK; 2 Wolfson School of Mechanical, Electrical and Manufacturing Engineering, Loughborough University, Loughborough, UK; 3 Labosport, Unit 3 Aerial Way, Hucknall, UK

**Keywords:** Mechanical engineering, Physiology

## Abstract

Mechanical testing by sporting governing bodies ensures artificial turf surfaces conform with performance standards. Rotational traction is measured using two equivalent devices: a rotational traction tester (RTT) and a lightweight rotational traction tester (LRTT). The devices differ in target rotational velocity; 72 deg/s for the RTT and 30 deg/s for the LRTT. The purpose of this study was to investigate the influence of rotational velocity on peak torque during rotational traction testing. An automated rotational traction tester examined nine rotational velocities between 10 and 210 deg/s, and ten artificial turf surface systems with a range of performance infill materials, infill depths and carpet specifications. Rotations at 10 deg/s produced the lowest peak torques on nine of the ten surfaces. Infill materials with intrinsic viscoelastic properties produced significantly higher peak torques at higher rotational velocities, whereas less elastic infill materials saw no significant increase in peak torque. A mean difference in peak torque of 2.6 Nm was found between the target velocities of the RTT and LRTT. The results support the synchronisation of target velocities for the RTT and LRTT. During standards testing, trials completed below a particular velocity should be repeated to negate velocity effects on peak torque.

## Introduction

Artificial turf surfaces are growing in popularity due to their greater durability and extended play hours compared to natural turf^1^. Governing bodies such as the Fédération Internationale de Football (FIFA) and World Rugby have developed testing programmes to ensure the safety and performance of artificial turf surfaces^2–4^. Rotational traction is one of the critical player-surface interaction measurements. Increased traction improves performance levels; however, excessive traction may increase the risk of lower limb injuries such as sprains and ligament damage^5, 6^. FIFA specify two acceptable upper and lower limits for rotational traction depending on the intended use of the surface: FIFA Quality Pro and FIFA Quality. To pass the highest quality specification (FIFA Quality Pro), a surface’s mean peak torque must fall between 30 and 45 Nm, while wider limits of 25–50 Nm are permitted for recreational level surface systems (FIFA Quality)^3^.

FIFA specify two devices for measuring rotational traction, a heavyweight tester (RTT) and a lightweight tester (LRTT)^3^. Both devices follow similar operating procedures: loading and manually rotating a circular, studded test foot at a target velocity on the surface, and recording the peak torque generated during the rotation. The target rotational velocities and magnitudes of rotation specified for the two devices differ: the LRTT target is 120° of rotation at 30 deg/s, and the RTT target is at least 45° of rotation at 72 deg/s. Previous studies have found a systematic bias in peak torque measured by the two devices, with the LRTT underreading the RTT by 2.2 Nm on average^7^. No conclusive reason for this disparity was found, however, the difference in rotational velocity has been suggested as a potential cause^7^. Further questions were then raised as to what effect rotational velocity may have on the traction response of artificial turf surface systems.

Recent studies have suggested using additional instrumentation during rotational traction testing to record the velocity achieved during a rotation, with the aim of improving reproducibility. However, the effect of rotational velocity on peak torque remains unknown^8^. Wannop et al.^9^ studied the relationship between movement speed and traction using an automated device, both for translational and rotational movements. The study found a positive linear relationship between movement speed and translational traction for velocities between 50 and 200 mm/s and a normal load of 580 N. No such effects were seen when analysing rotational traction. Testing was completed at three rotational velocities (30 deg/s, 60 deg/s and 90 deg/s) and a normal load of 580 N. Three different football boot outsoles were tested on one artificial turf surface using cryogenic styrene-butadiene rubber (SBR) as the performance infill. Wannop et al. suggested that slower rotational velocities may be suitable for rotational traction testing, as no effect on peak torque was observed in their results. Wannop et al.’s. test methods varied from FIFA’s test methodologies, with differences in load, stud geometry and stud configuration. Such variables are known to affect traction response^9–14^, indicating the conclusions of their study may not be valid for FIFA test devices. The only study found to evaluate the effect of rotational velocity on peak torque when using a FIFA standard RTT was conducted by Webb et al.^10^. Webb^10^ evaluated the effect of three different rotational velocities (24 deg/s, 48 deg/s and 72 deg/s) on peak torque, for an SBR infilled surface system; peak torque decreased at the lowest rotational velocity, however no difference in peak torque was found between 48 and 72 deg/s.

The use of SBR as a performance infill material faces a potential ban from the European Chemical Agency (ECHA), due to SBR’s classification as an intentionally added microplastic^15^. Styrene-butadiene rubber granules are made from end-of-life tyres and contain polycyclic aromatic hydrocarbons (PAH) among other elements which potentially cause harm to human health and the natural environment^16^. A recent study estimated that artificial turf surfaces are one of the largest emitters of microplastics across Europe, with pitches losing approximately 72,000 tonnes of performance infill to the local environment each year^17^. This has led to an increase in the market of alternative surface systems, with organic performance infill materials such as cork and pine woodchip, and new non-filled artificial turf surfaces becoming more widely used. Guidance from the Sports and Play Construction Association (SAPCA) states that organic infilled and non-filled surfaces provide an environmentally friendly alternative to SBR infilled pitches; however, many organic performance infills require lengthy transportation from source, arguably reducing their sustainability credentials^18^.

The total cost of installing an organic or non-filled surface system varies less than 20% in comparison to an SBR infilled surface; however, the performance characteristics of organic and non-filled surface systems has been questioned^17^. Performance infills can be likened to a granular material due to their assembly of particles; in general, the mechanical behaviour of granular materials, i.e., shear strength and compression, depends on the size and shape of the particles, their packing arrangement, associated pore spaces, and for many materials also the degree of saturation^19^. Reports suggest that some organic performance infills of low particle density are prone to floating or waterlogging in very heavy rain, whilst also being susceptible to freezing in colder conditions^18^, however experience and published research into many of the organic infilled and non-filled surfaces systems is currently lacking.

To ensure current test methods remain suitable, it is paramount to understand how rotational velocity affects traction across a range of different surface constructions. To date, no study has examined the effect of rotational velocity on peak torque using FIFA’s specified test devices; likely due to the challenges in producing controlled, repeatable rotations at a target velocity when using manually operated devices. This constitutes a large gap in knowledge given the current disparity between target velocities of both FIFA approved test devices, and the range of new performance infill materials and surface systems coming to market.

The primary aim of this study was to determine the effect of rotational velocity on peak torque across a range of surface systems. An automated rotational traction tester (ARTT) controlled the rotational velocity during testing, replicating and extending on the rotational velocities applied by manual devices. Analysing the effect of rotational velocity can inform future developments in artificial turf testing procedures, especially in standardised test methodologies set by governing bodies such as FIFA. The secondary aim was to investigate how different surface systems and performance infill materials respond to changes in rotational velocity. The impending ban on SBR as a performance infill material means it is more crucial than ever to understand the behaviour of different performance infill materials, ensuring safety and performance in future artificial turf developments.

## Methodology

### Surface systems

**Figure 1:**
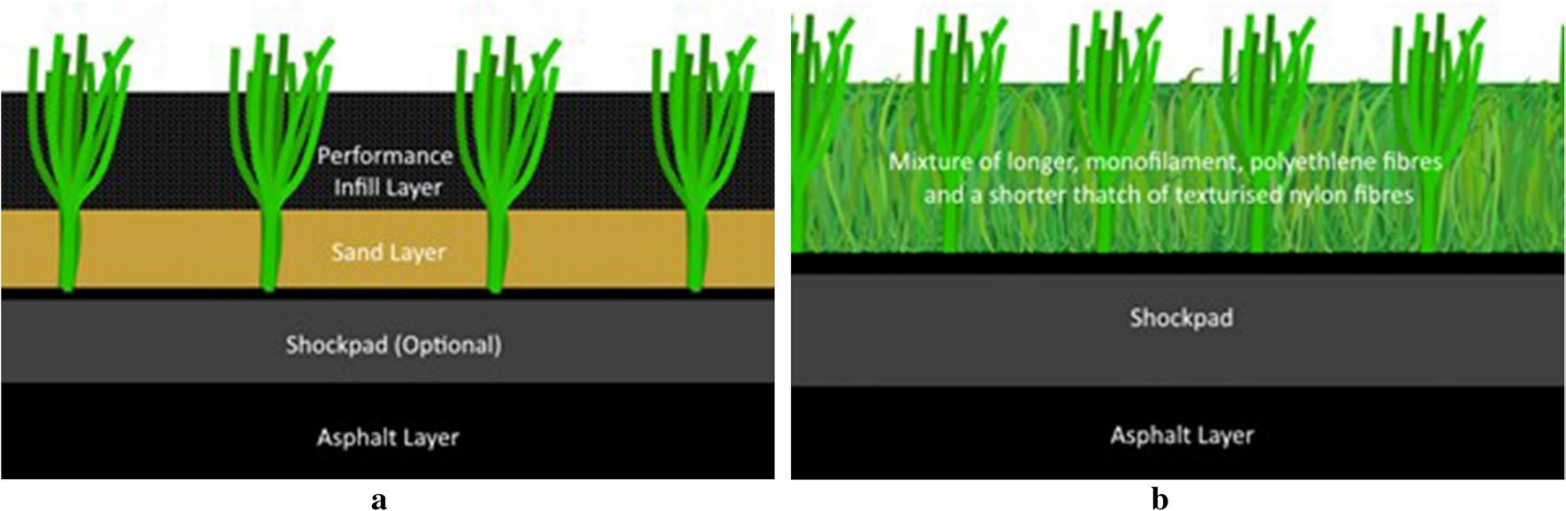
(**a**) Schematic diagram of an infilled artificial turf surface. (**b**) Schematic diagram of a non-filled artificial turf surface.

This study tested ten artificial turf surface systems: eight infilled surfaces using four different infill materials and two non-filled surfaces (Table 1). The common construction profile of the infilled, and non-filled artificial turf surfaces is shown in Fig. 1a and b respectively. For this study, the asphalt layer was replicated using a concrete laboratory floor at Loughborough University. The surface systems used in the study were selected to represent a broad range of infill materials, surface specifications and carpet pile heights currently available in the artificial turf industry.

**Table 1:** Construction properties of the eight infilled surfaces used for the study.

Surface Name	Pile Height (Carpet Specification)	Pile Weight (g/m^2^) (%)	Total Infill Depth (mm)	Performance Infill Material (size range) [Supplier]	Performance Infill Mass (kg/m^2^)	Performance Infill Depth (mm)	Stabilising Infill* (size range)	Stabilising Infill Mass (kg/m^2^)	Sand Infill Depth (mm)	Shock Pad Thickness (mm) [Manufacturer]
SBR_50a	50 mm (Tiger Turf Atomic Pro)	1249 ± 10	39	SBR (0.8–2.5 mm) [Murfitt Industries]	20.0	20	Sand (0.2 –0.7 mm)	15.0	19	11 [Alveo 3011]
SBR_50b	50 mm (CC Grass Stemgrass-5004B120-BL)	1099 ± 10	30	SBR (0.8–2.5 mm) [Murfitt Industries]	16.0	23	Sand (0.2 –0.7 mm)	10.0	7	None
SBR_60	60 mm (Tiger Turf Atomic Pro)	1431 ± 10	44	SBR (0.8–2.5 mm) [Murfitt Industries]	17.0	30	Sand (0.2 –0.7 mm)	20.0	14	None
EPDM_60	60 mm (SIS Turf Xtreme Ultra)	1618 ± 10	48	Recycled EPDM (1.3–3.2 mm) [SIS Pitches]	15.0	25	Sand (0.2 –0.7 mm)	25.0	23	14 [Revosport]
CORK_40	40 mm (SIS Turf Xtreme Ultra)	N/A ± 10	30	Cork (1.0–2.0 mm) [Amorim]	1.7	16	Sand (0.2 –0.7 mm)	17.0	14	11 [Alveo 3011]
CORK_60	60 mm (Tiger Turf Atomic Pro)	1431 ± 10	48	Cork (1.0–2.0 mm) [Amorim]	4.0	34	Sand (0.2 –0.7 mm)	17.0	14	None
PINE_50	50 mm (Tiger Turf Atomic Pro)	1249 ± 10	34	Pine Woodchip (0.5–3.5 mm) [BrockFILL]	4.9	18	Sand (0.2 –0.7 mm)	19.4	16	23 [Brock PBEURO23]
PINE_60	60 mm (Tiger Turf Atomic Pro)	1431 ± 10	44	Pine Woodchip (0.5–3.5 mm) [BrockFILL]	6.5	24	Sand (0.2 –0.7 mm)	24.2	20	23 [Brock PBEURO23]
NON-PRO	28 mm (Greenfields Slide Max Pro NF)	2.62 ± 10	N/A	N/A	N/A	N/A	N/A	N/A	N/A	20 [Schmitz foam ProPlay-Sport20]
NON-REG	28 mm (Greenfields Max NF)	3.65 ± 10	N/A	N/A	N/A	N/A	N/A	N/A	N/A	20 [Schmitz foam ProPlay-Sport20]

Surface name is denoted by the performance infill material, carpet manufacturer and pile height.

*All stabilising sand was Garside 2EW.

**Figure 2:**
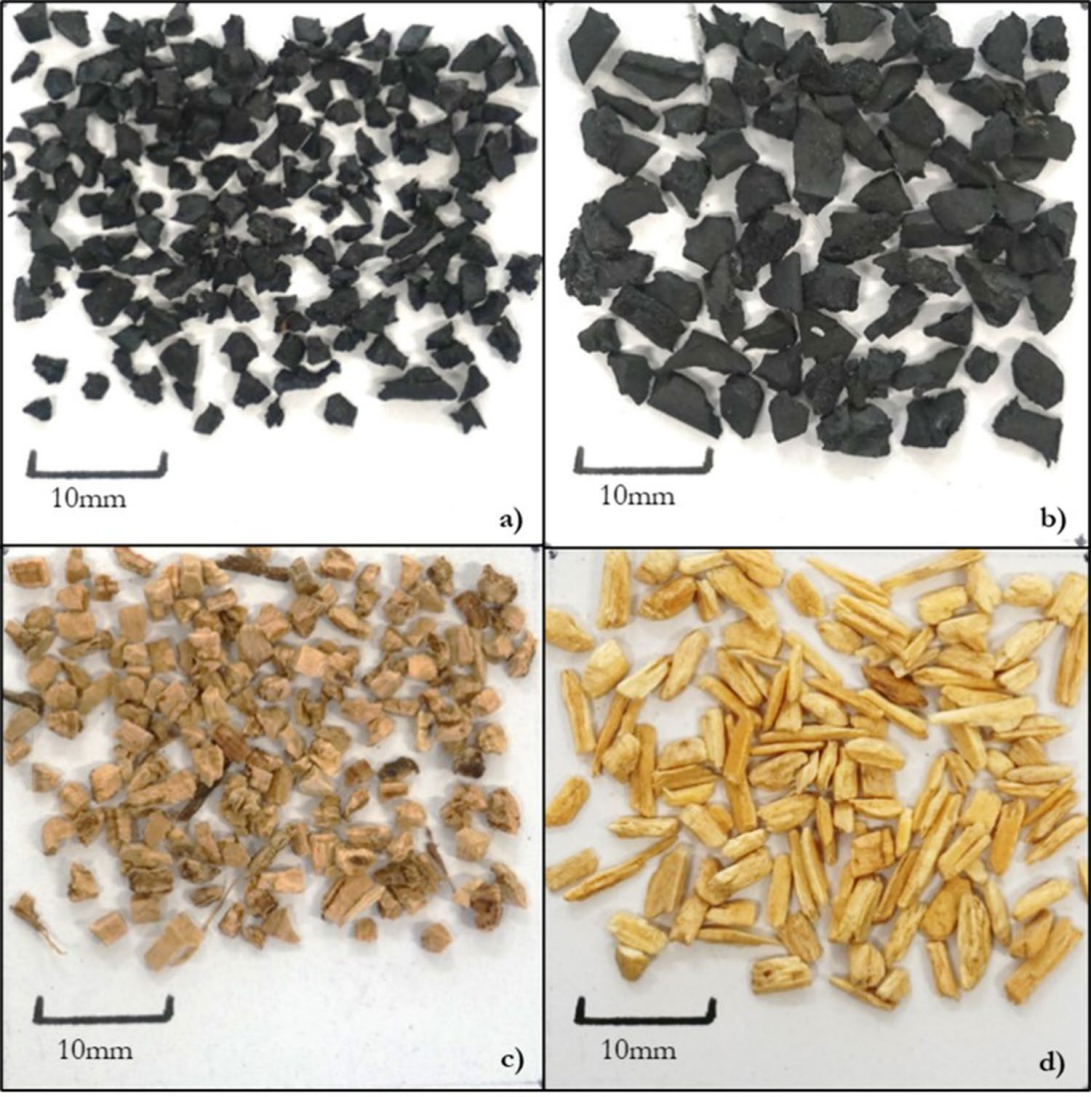
The performance infills used in the study, (**a**) SBR, (**b**) Recycled EPDM, (**c**) Cork, (**d**) Pine woodchip.

Each surface was carefully prepared following the guidelines in the FIFA Quality Programme for Artificial Turf^3^. The infilled surfaces comprised a 1 m^2^ section of carpet, first filled with a layer of stabilising silica sand, and then topped with a respective performance infill upper layer—as per industry practice. The infills used during the study are shown in Fig. 2. Each surface was prepared in accordance with the manufacturer’s specifications where possible. The non-filled surfaces were both 5 m × 1 m rolls of carpet. When required, a shock pad was placed underneath the surface system, in line with the manufacturers specifications. Prior to testing, the infilled surfaces were raked and rolled 50 times with a studded roller before nine infill depth measurements were taken across the surface. The non-filled surface was only raked prior to testing.

### Test device

**Figure 3:**
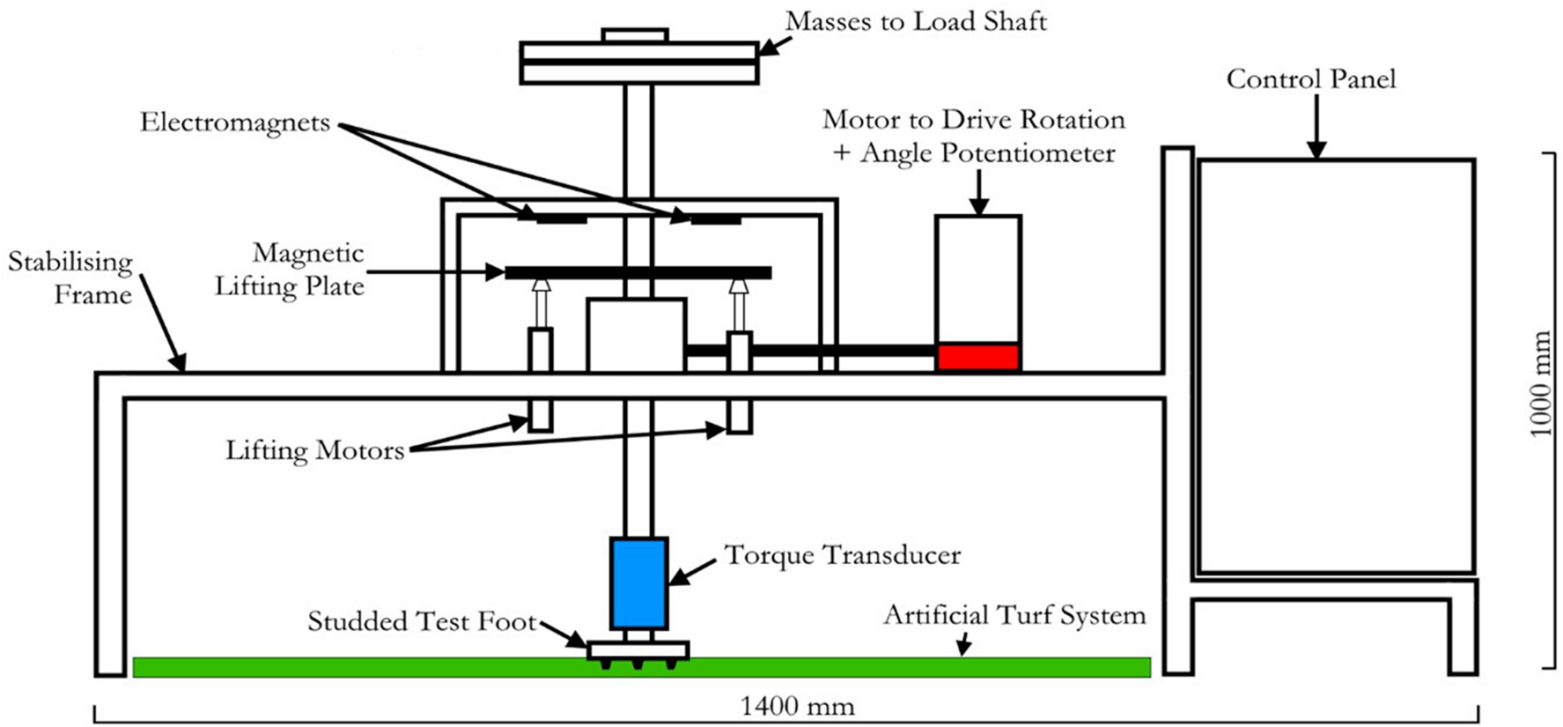
A schematic diagram of the ARTT developed at Loughborough University.

An automated rotational traction tester (ARTT) was used to conduct the rotational traction testing on each surface (Fig. 3). The ARTT was purpose built at Loughborough University as a research tool for artificial turf surfaces and can reproduce the test conditions in the FIFA Handbook of Test Methods for Artificial Turf^2^. A circular studded test foot (150 mm in diameter containing six studs 13 mm in length, equally spaced at a radius of 46 mm from the centre) was loaded with 450.2 ± 20.0 N using static masses^7^. Electromagnets suspended the test foot 60 ± 5 mm above the surface, enabling full stud penetration into the surface when the test foot was dropped vertically. The ARTT contained a strain gauge torque sensor to record the torque generated during a rotation, with a resolution of 0.01 Nm, accuracy of ± 0.1 Nm. A Hall sensor potentiometer recorded changes in angle, at a resolution of 0.5° and an accuracy of ± 1.0°. Angle and torque data was sampled at 1000 Hz using LabView software (National Instruments, Austin, USA). The ARTT’s instrumentation records changes in voltage. Sensitivity values, calculated during calibration of the test device, convert the outputs to respective torque and angle values. A programmable motor drives the test foot’s rotation, enabling rotations up to 220°, rotational velocities of 0–225 deg/s and accelerations of up to 2140 deg/s^2^.

### Data collection

Data collection occurred over a two-week period, with each surface system allocated one day for testing. Data collection followed the protocols outlined in the FIFA Handbook of Test Methods^2^. Nine trials were conducted for each surface sample and rotational velocity. One trial consisted of dropping the test foot vertically onto the surface and allowing the test foot to settle, before rotating the test foot 180° at the desired rotational velocity. After the rotation, the test foot returned to its original drop height and start position. The surface was manoeuvred below the test foot and a new location tested. Test locations were at least 100 mm from the edge of the surface, and 100 mm from a previous test location (edge of test foot to edge of test foot)^2^. Once nine trials were complete, the surface was reconditioned (raking, 50 rolls using a studded roller and 9 infill depths measurements where appropriate) before moving to the next rotational velocity. The same researcher conducted each reconditioning. Nine rotational velocities were analysed: 10 deg/s, 30 deg/s (the LRTT’s target rotational velocity^2^), 50 deg/s, 72 deg/s (the RTT’s target rotational velocity^2^, 90 deg/s, 110 deg/s, 130 deg/s, 150 deg/s and 210 deg/s. To ensure the study incurred no bias, the order of velocities tested was randomised for each surface sample, using an online calculator^20^. Rotational acceleration was constant throughout testing, set at 2140 deg/s^2^.

### Data processing

Data from each trial was stored as a .csv file. Raw data was filtered in a custom MATLAB script (R2021b, The MathWorks, Natick, NJ, USA) using a 2nd order, low pass Butterworth filter (20 Hz)^7^. The MATLAB script calculated relevant outputs such as peak torque, angle of peak torque and mean rotational velocity for each trial. Rotational velocity was calculated by numerically differentiating the change in angle throughout the full rotation. Trials for each combination of surface system and rotational velocity were processed together, with the key data stored in a single excel file. Mean and standard deviation values were then calculated in Microsoft Excel.

### Statistical analysis

All statistical analysis was conducted in Microsoft Excel. To evaluate whether significant differences existed between the peak torques achieved at different rotational velocities, a one-way ANOVA was conducted on each surface and rotational velocity. To determine where significant differences existed, a paired t-test was conducted. To correct for the increased risk of Type 1 errors when reusing a dataset, a Bonferroni correction was used^21, 22^. Therefore, α level of significance was set at 0.001.

## Results

**Figure 4:**
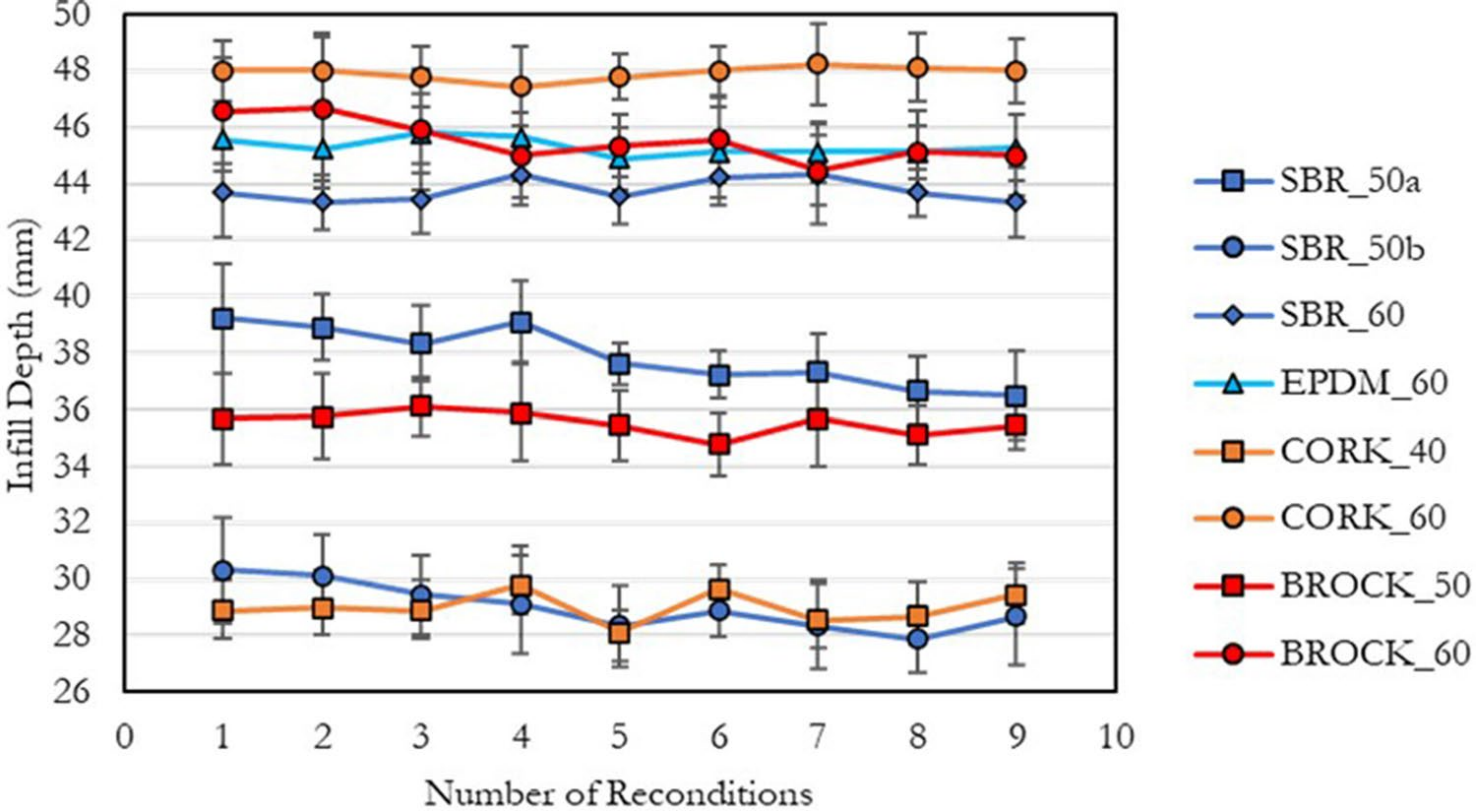
Mean (± standard deviation) infill depth for each infilled surface; recorded after every surface reconditioning.

For all surfaces, the maximum difference in mean infill depth between reconditions was less than 3 mm; showing good surface consistency throughout the study. No surface required additional performance infill to maintain infill depth during the study (Fig. 4).

**Figure 5:**
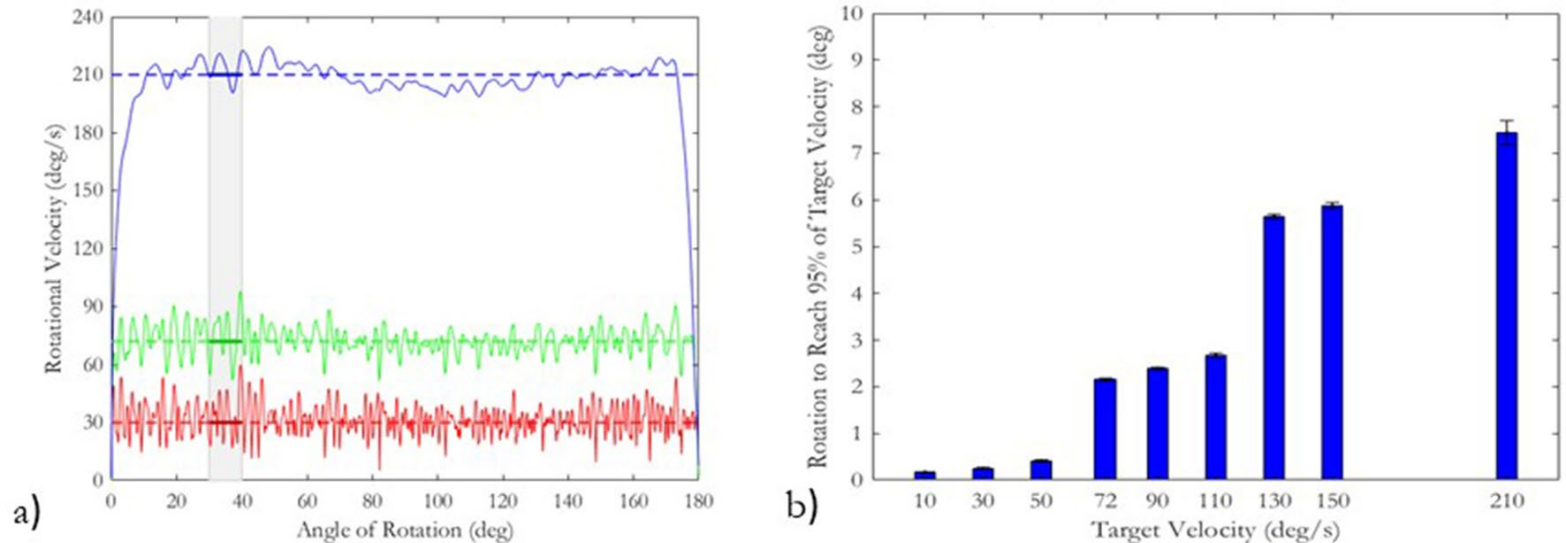
(**a**), Example velocity profiles for rotations at 30 deg/s, 72 deg/s and 210 deg/s. Highlighted in the plot is an area between 30° and 40°, the common range of angles where peak torque was achieved during a rotation. (**b**), The angle of rotation at which the ARTT reached 95% of each target velocity.

Examples of three velocity-angle profiles from the ARTT are shown in Fig. 5a. The rotations shown are 30 deg/s (FIFA’s LRTT target velocity), 72 deg/s (FIFA’s RTT target velocity) and 210 deg/s. Peak torque commonly occurs between 30° and 40° of rotation^9^, highlighted in grey in Fig. 5a. The angle of rotation to reach 95% of each target velocity is shown in Fig. 5b. The ARTT reached 95% of the target rotational velocity in under 2.5 s, for target rotational velocities up to 90 deg/s. At higher velocities such as 210 deg/s, 95% of the target velocity was not reached until degrees 7.4° of rotation. Between 30° and 40° of rotation, the rotational velocity was consistent with the target velocities input into the test device.

**Figure 6:**
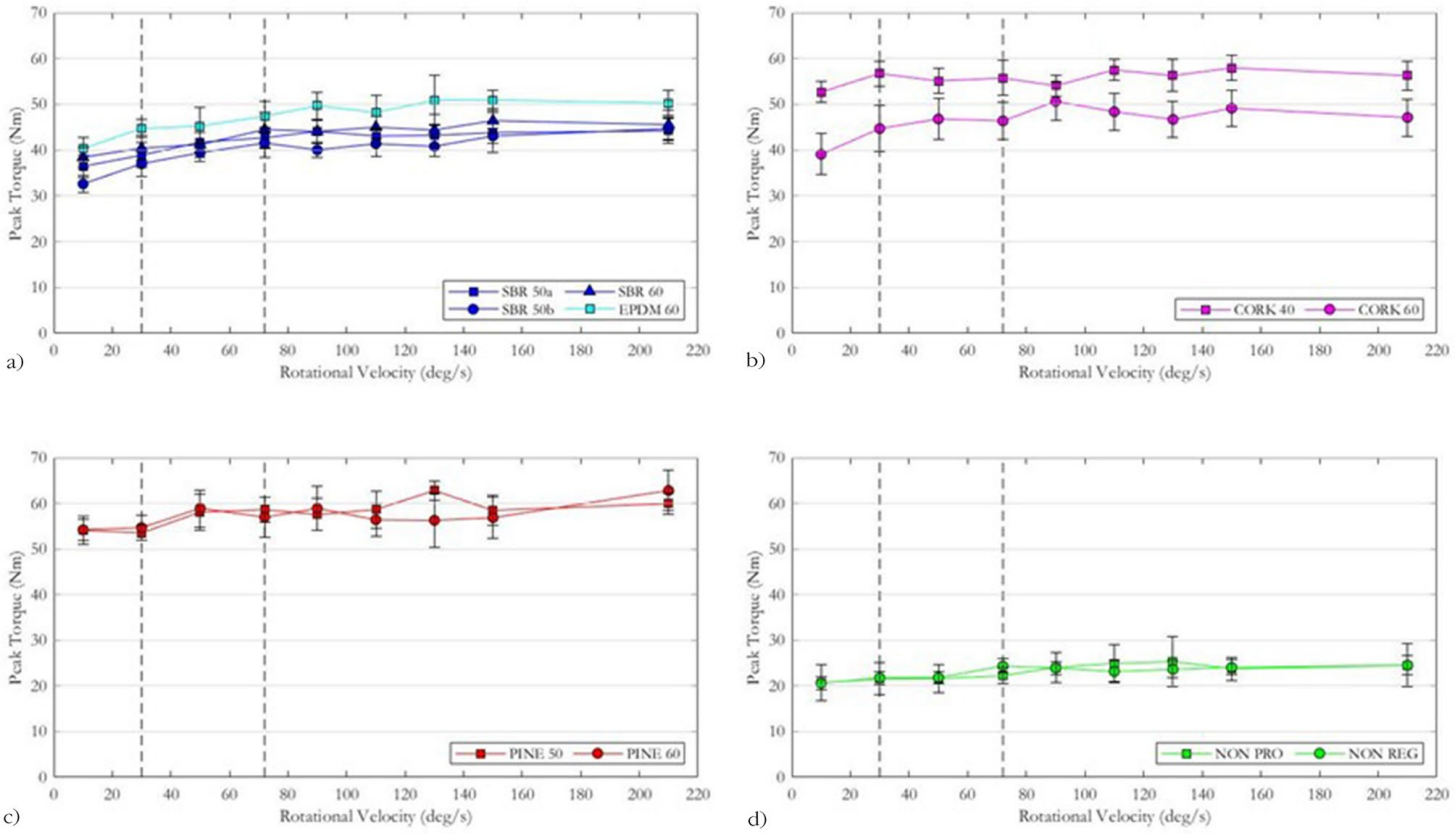
The relationship between peak torque and rotational velocity for the (**a**) polymeric-, (**b**) cork-, (**c**) pine woodchip- and (**d**) non-filled surfaces. The target rotational velocities for the LRTT and RTT are shown in black dashed lines.

The relationship between peak torque and rotational velocity for the SBR and EPDM (rubber/polymeric) infilled surfaces is shown in Fig. 6a. All surface systems followed a similar trend, showing an increase in peak torque from 10 deg/s to between 72 and 90 deg/s, followed by a plateau at higher rotational velocities. The increase in peak torque from 10 deg/s to the largest peak torque value was 7.7 Nm (21.1%) for SBR_50a, 8.0 Nm (20.8%) for SBR_50b, 12.1 Nm (37.1%) for SBR_60, 10.6 Nm (26.3%) for EPDM_60.

The relationship between peak torque and rotational velocity for both cork-filled surfaces is shown in Fig. 6b. The two cork-filled surfaces exhibited a different relationship between peak torque and rotational velocity. The CORK_40 surface demonstrated little effect of rotational velocity on peak torque, producing peak torque values ranging from 52.7 to 57.9 Nm, a 5.2 Nm increase (9.9%). In contrast, the CORK_60 surface followed a similar trend to the polymeric infilled surfaces; lowest peak torque values were measured at 10 deg/s (39.1 Nm) before reaching a maximum plateau value of 50.6 Nm at 90 deg/s, an increase of 11.5 Nm (29.4%).

The relationship between peak torque and rotational velocity for the two pine-woodchip infilled surfaces is shown in Fig. 6c. On both surfaces, there was a small increase in peak torque with rotational velocity. For the PINE_50 surface, peak torque values ranged from 53.5 Nm at 30 deg/s to 62.8 Nm at 130 deg/s, a 9.5 Nm increase (17%). For PINE_60 surface the peak torque values increased from 54.2 Nm at 10 deg/s to 62.8 Nm at 210 deg/s, an 8.6 Nm increase (16%).

The relationship between peak torque and rotational velocity for both non-filled surfaces is shown in Fig. 6d. On the NON_PRO surface, peak torque values increased from 20.7 Nm at 10 deg/s to 25.3 Nm at 130 dg/s, a 4.7 Nm increase (22.5%). For the NON_REG surface, peak torque increased from 20.6 Nm at 10 deg/s to 24.5 Nm at 210 deg/s, a 3.9 Nm increase (19%).

**Figure 7:**
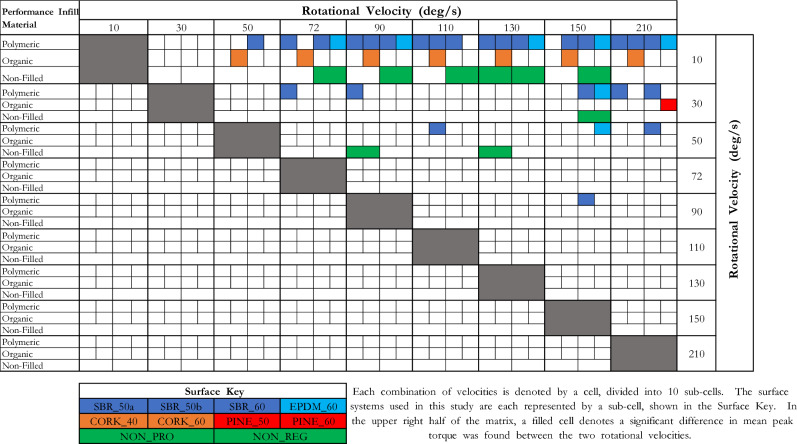
Comparison matrix to show where significant differences in mean peak torque existed between two rotational velocities, on the same surface.

The combination of rotational velocities and surface systems that produced statistically significant differences in peak torque values is shown in a comparison matrix (Fig. 7). A description of how to use the comparison matrix is included in the figure. The right-hand side of the comparison matrix indicates where significant differences exist between two mean peak torques recorded at different rotational velocities, for the same surface; the left side of the matrix shows the effect size for each velocities and surface combination.

Visually, the comparison matrix reinforces that the influence of rotational velocity on peak torque is dependent on the range of rotational velocities tested and the design of the surface system. Most of the significant differences were found between 10 - 50 deg/s, and a higher rotational velocity (48/49 significant differences). Only one significant difference was found comparing values of 72 deg/s and greater, across all surfaces (SBR_60 at 90 deg/s and 150 deg/s).

Higher peak torque values were found at 72 deg/s (RTT target velocity) compared to 30 deg/s (LRTT target velocity) on all surface systems except CORK_40. The range of differences in peak torque between 72 and 30 deg/s was -1.0 Nm to + 5.7 Nm, with a mean difference of + 2.6 Nm. Only one surface system produced significantly higher peak torque values at 72 deg/s compared to 30 deg/s (SBR_50a). Relatively few significant differences were found on the organic infilled surfaces; with only the CORK_60 surface producing more than one significant difference between two rotational velocities.

## Discussion

The aim of this study was to investigate the effect of rotational velocity on peak torque across a range of different artificial turf surface systems. Current differences in target rotational velocity of FIFA’s two rotational traction testers^3^ highlights the need to better understand the relationship between peak torque and rotational velocity. The results of the study have shown the effect of rotational velocity on peak torque is dependent on the design of the surface system and the velocity achieved during rotation.

### Effect of rotational velocity on peak torque

Peak torque appears to be most affected by low rotational velocities; 10 deg/s produced the lowest peak torque values across nine of the ten surface systems tested. Across all the surface systems tested, 35/49 (71.4%) of the significant differences in peak torque were found between 10 deg/s and a higher rotational velocity. At higher velocities, the effect of rotational velocity on peak torque appears to plateau. At velocities higher than 72 deg/s, only one significant difference was found across all surfaces (SBR_60 comparing 90 deg/s and 150 deg/s), with no significant differences found when comparing velocities of 110 deg/s and higher.

This study’s findings agree with those of Webb et al.^10^; who reported that rotations at 24 deg/s produced lower peak torque values compared to 48 deg/s and 72 deg/s, with no difference in peak torque between rotations at 48 deg/s and 72 deg/s for an SBR infilled surface. The current study improved on the methodologies published by Webb et al. using an automated rotational traction tester rather than a manual device. Manually rotated test devices are likely to be unreliable at producing controlled, repeatable rotations to a specific target velocity. Additionally, the study did not publish details of the surface system, the number of trials or reconditioning procedures used; all of which have been published for this study. Wannop et al.^9^ found no effect of rotational velocity on peak torque for a cryogenic-SBR infilled surface. The lowest rotational velocity tested by Wannop et al. was 30 deg/s; the results of the current study suggest that had a lower velocity been included, an effect on peak torque may have been obtained. Furthermore, Wannop et al. completed rotational traction tests using three football boot outsoles at loads of 580 N; thus, comparing the results found by Wannop et al. to the current study is challenging^9–14^.

The current study comprehensively analysed the effect of rotational velocity on peak torque, using a broad range of rotational velocities from 10 deg/s to 210 deg/s. Previously, the minimum and maximum rotational velocities investigated were 24 deg/s and 90 deg/s, respectively. Although manual operators are extremely unlikely to perform rotations at velocities close to 210 deg/s, the results of the study provide a first insight into how peak torque is affected at such velocities. The impact of low rotational velocities on peak torque was also highlighted in this study and has several implications to the testing industry; detailed in Sect. 5.3.

### Effect of surface system on peak torque

For infilled surfaces, both infill material and infill depth influenced the effect of rotational velocity on peak torque. All four polymeric infilled surfaces followed the same trend: the lowest peak torque value was found at 10 deg/s, with peak torque increasing until reaching a plateau at approximately 72 deg/s. The pine woodchip infilled surfaces were largely unaffected by increases in rotational velocity, with only one significant difference in peak torque found on either surface (Fig. 7). The two cork infilled surfaces provided contrasting relationships for increasing rotational velocities. The CORK_40 surface produced no significant differences in peak torque at all velocities tested; whereas the CORK_60 surface followed a similar trend to the polymeric infilled surfaces, with rotations at 10 deg/s producing the lowest peak torques, followed by a plateau at higher velocities. For non-filled surfaces, three significant differences in peak torque values were found on the NON_PRO carpet, whereas six differences in peak torque were found between velocities on the NON_REG carpet. The results, therefore, show that the design of the surface system affects the relationship between rotational velocity and peak torque.

This study is the first in literature to investigate the effect of rotational velocity on a range of different surface systems. To date, only SBR has been used as a performance infill material when assessing the effect of rotational velocity; Webb et al., used regular SBR, while Wannop et al., used cryogenic SBR^9, 10^. Of the performance infill materials used in this study (SBR, recycled EPDM, cork and pine woodchips), only the peak torque values achieved on pine woodchip infilled surfaces were consistently unaffected by changes in rotational velocity. The reason for this is likely due to the performance infill material. The pine woodchip particulate^23^, is considered a material of low elasticity^24, 25^, whereas SBR, EPDM and cork are materials that exhibit greater elasticity and strong viscoelasticity, i.e., high strain rate dependency of the stress strain properties^1, 26–29^. Campbell et al., state the elasticity of a material is also an important parameter when determining the type and magnitude of shear resistance in a granular material^30, 31^. Artificial turf surface systems are not purely granular in nature due to the carpet’s polyethylene fibres which are known to reduce the mobility and increase the shear resistance of the infill, i.e., provide a confining effect^32^. It is hypothesised that the viscoelastic nature of SBR, EPDM and cork contribute to the increasing peak torque values observed at increasing rotational velocities.

The design of the surface system also affects how peak torque varies with changes in rotational velocity. No significant differences between peak torque values were seen on the 40 mm fibre length carpet and cork system (CORK_40), whereas for the 60 mm fibre length system (CORK_60) peak torque values increased from lower rotational velocities before plateauing, like the trend seen on the polymeric infilled surfaces. Like rubber, cork is considered a viscoelastic material^26, 27^. On the CORK_40 surface, it is thought that the stud may have penetrated through to the sand layer, creating a different response to changes in rotational velocity. The total depth of performance infill material was 16 mm, while the (standard) stud length used in the study was 13 mm^2^. Therefore, it is plausible that under the normal compressive load applied the studs may penetrate the sand layer, increasing resistance. Laboratory shear tests have demonstrated silica sand has a much higher shear strength and lower compressibility relative to SBR^33^. This may help explain the greater peak torques observed on the shorter pile CORK_40 surface system relative to the CORK_60 system, and different response to changes in rotational velocity. However, this possible effect is hard to observe without better understanding the compression behaviour of the performance infill layer.

Both the non-filled surfaces experienced different peak torque responses to changes in rotational velocity. The NON_REG surface showed significant differences in peak torque at primarily low velocities (10 deg/s), whereas the NON_PRO surface did not follow this trend. The reason for this remains unclear. The two non-filled surfaces appear similar in design, each containing a combination of polyethylene fibre shapes with a main pile height of 28 mm and a thatch (root) pile. However, the increased pile weight (Table 1) and denser thatch zone in the NON_REG surface system appear greater influenced by changes in rotational velocity, especially at low rotational velocities such as 10 deg/s.

A range of different performance infill materials are currently being introduced into the artificial turf market, partly due to a potential ban on the sale of SBR^15^ and increasing focus on sustainability within the industry. This study has provided a comprehensive initial investigation into how different surface systems respond to changes in rotational velocity, for the specific rotational traction test applied to new products in the laboratory and field installations^2–4^. The broadening range of performance infill materials used within the industry strongly supports the hypothesis that future work should focus on understanding the intrinsic material properties of new polymeric-, organic- and non-filled surfaces. Better understanding how these surfaces may perform under different loading conditions is paramount to the safety and performance of future artificial turf systems. Furthermore, there is a need to better understand how traction is developed during a rotational traction test. Greater comprehension of the mechanisms that generate traction resistance should aid the understanding of which infill material characteristics (in combination with the carpet system) are most relevant for producing an “optimal surface system.”

### Implications for industry

The outcomes of this study highlight key implications for rotational traction testing standards detailed in the FIFA Handbook of Test Methods^3^. There is a need to synchronise the target rotational velocities of FIFA’s two test devices, as lower velocities appear to influence peak torque. Furthermore, with the additional instrumentation to measure change in angle suggested by Cole et al., trials falling below a particular rotational velocity should be repeated to negate the effect of low rotational velocities on peak torque^8^. When comparing the peak torques achieved at 30 deg/s and 72 deg/s (FIFA’s target velocities for the LRTT and RTT respectively), rotations at 72 deg/s produced peak torque values 2.6 Nm greater, on average, than rotations at 30 deg/s. The difference presented in the current study agrees with previously published literature comparing peak torque measurements using an RTT and LRTT, where a systematic bias of 2.2 Nm was found between test devices^7^. When considering rotational traction testing standards, the target rotational velocity for both devices should be a compromise between a velocity high enough to negate the effect of rotational velocity on traction, but also a velocity that is achievable by manual operation, without jeopardizing the overall testing procedure. Alongside synchronising the rotational velocities, a lower limit for rotational velocity should be set for all trials, to remove the effect of low rotational velocities (10 deg/s) on peak torque values. This becomes more paramount when analysing the peak torque values of alternative artificial turf surface systems surfaces. Figures 6, 7, and 8 show the peak torques recorded for cork, pine woodchip and non-filled surfaces. The peak torque values sit close to the 25–50 Nm limits for rotational traction in the FIFA Quality standard of turf^2^. The results and subsequent implications of this study are an initial step in ensuring rotational traction test methodologies remain accurate for laboratory and field testing, across a range of different surface systems.

## Limitations

The study’s main limitation was the ability of the motor to accelerate when driving the rotation of the ARTT’s test foot. At 210 deg/s, the ARTT reached 95% of its target velocity at 7.4° of rotation. For rotational traction testing, this is not such a large problem as peak torque is commonly achieved between 30 and 40° of rotation due to stud overlap mechanics^8, 9^. Consequently, the ARTT’s test foot was rotating at the target velocity well before peak torque was achieved. However, in human-subject studies, it is reported the foot rotates 18.1° ± 12.3° during a stop and turn movement^34^. The smaller rotations seen in human-subject studies indicate the ARTT may not be capable of accurately reproducing human-specific movements. Future automated test devices should aim to include a motor with increased acceleration and velocity capabilities, helping to establish greater scientific comprehension of the traction experienced by an athlete when interacting with an artificial turf system.

## Conclusion

The study was the first in literature to comprehensively analyse the effect of rotational velocity on peak torque measurements across a range of different artificial turf surface systems. The results showed peak torque was affected by two variables: the rotational velocity used during testing and the design of the artificial turf surface system. Rotations at 10 deg/s produced the lowest peak torque values on all surfaces except one. At velocities higher than 110 deg/s, no significant differences in peak torque were seen on any surface. Viscoelastic performance infill materials such as SBR, EPDM and cork were most affected by changes in rotational velocity, while the pine woodchip infill was largely unaffected by changes in rotational velocity. A mean difference of 2.6 Nm was found between rotations at 72 deg/s (target velocity of the RTT) and 30 deg/s (target velocity of the LRTT). A recommendation has been made to synchronise the target velocities of the two FIFA test devices, whilst also recording velocity and repeating trials which fall below a particular threshold during manually operated field testing.
